# QTL Analysis for Transgressive Resistance to Root-Knot Nematode in Interspecific Cotton (*Gossypium* spp.) Progeny Derived from Susceptible Parents

**DOI:** 10.1371/journal.pone.0034874

**Published:** 2012-04-13

**Authors:** Congli Wang, Mauricio Ulloa, Teresa R. Mullens, John Z. Yu, Philip A. Roberts

**Affiliations:** 1 Department of Nematology, University of California Riverside, Riverside, California, United States of America; 2 United States Department of Agriculture-Agricultural Research Service, Western Integrated Cropping Systems Research Unit, Shafter, California, United States of America; 3 United States Department of Agriculture-Agricultural Research Service, Southern Plains Agricultural Research Center, College Station, Texas, United States of America; East Carolina University, United States of America

## Abstract

The southern root-knot nematode (RKN, *Meloidogyne incognita*) is a major soil-inhabiting plant parasite that causes significant yield losses in cotton (*Gossypium* spp.). Progeny from crosses between cotton genotypes susceptible to RKN produced segregants in subsequent populations which were highly resistant to this parasite. A recombinant inbred line (RIL) population of 138 lines developed from a cross between Upland cotton TM-1 (*G. hirsutum* L.) and Pima 3–79 (*G. barbadense* L.), both susceptible to RKN, was used to identify quantitative trait loci (QTLs) determining responses to RKN in greenhouse infection assays with simple sequence repeat (SSR) markers. Compared to both parents, 53.6% and 52.1% of RILs showed less (*P*<0.05) root-galling index (GI) and had lower (*P*<0.05) nematode egg production (eggs per gram root, EGR). Highly resistant lines (transgressive segregants) were identified in this RIL population for GI and/or EGR in two greenhouse experiments. QTLs were identified using the single-marker analysis nonparametric mapping Kruskal-Wallis test. Four major QTLs located on chromosomes 3, 4, 11, and 17 were identified to account for 8.0 to 12.3% of the phenotypic variance (*R^2^*) in root-galling. Two major QTLs accounting for 9.7% and 10.6% of EGR variance were identified on chromosomes 14 and 23 (*P*<0.005), respectively. In addition, 19 putative QTLs (*P*<0.05) accounted for 4.5–7.7% of phenotypic variance (*R^2^*) in GI, and 15 QTLs accounted for 4.2–7.3% of phenotypic variance in EGR. In lines with alleles positive for resistance contributed by both parents in combinations of two to four QTLs, dramatic reductions of >50% in both GI and EGR were observed. The transgressive segregants with epistatic effects derived from susceptible parents indicate that high levels of nematode resistance in cotton may be attained by pyramiding positive alleles using a QTL mapping approach.

## Introduction

Host-plant resistance is highly effective in controlling crop loss from root-knot nematode (RKN) *Meloidogyne incognita* Kofoid and White (Chitwood) infection. Novel sources and enhanced levels of pathogen resistance are desirable for genetic improvement of crop plants. One source of enhanced resistance is that generated through transgressive segregation. Transgressive segregation is the phenomenon in which segregating hybrids exhibit phenotypes that are extreme or novel relative to the parental lines [1]–[2] and result from epistatic interactions between the genes assembled in novel combinations. Transgressive segregants for numerous traits have been reported, and can be derived from both intraspecific and interspecific crosses [2]–[4]. Breeding for optimal resistance must be based on selection of progeny with combinations of genes homozygous for resistance. However, a highly susceptible parent can contribute to nematode resistance via transgressive segregation [5]. These crosses can derive highly resistant lines, even when both parents have a susceptible phenotype. Such transgressive segregants can be used as improved resistance sources in crop breeding [5]–[6].

Transgressive segregation is one of the major selection sources in cotton (*Gossypium hirsutum* L.) for enhanced resistance to RKN, an important pest of cotton [7]. Wang et al. (2008) [5] reported that a segregant factor (*RKN2*) from one susceptible parent, *G. barbadense* L. Pima S-7 interacted with a major recessive gene *rkn1* from *G. hirsutum* Acala NemX [8] to produce a highly resistant phenotype in the interspecific cross Pima S-7 x Acala NemX. We also observed that a susceptible genotype Acala SJ-2 contributed to the level of resistance in some F_2:7_ (NemX x SJ-2) RI homozygous resistant lines [9]. Transgressive segregation also has been observed in other pathogen-cotton systems, such as for Verticillium wilt resistance in an F_2_ (resistant Pima S-7 x susceptible Acala 44) [10] and in an F_2:3_ family (*G. barbadense* H7124 x *G. hirsutum* XLZ1) [11], for Fusarium wilt resistance in the F_2_ or F_3_ populations (resistant Pima S-7 x susceptible Acala NemX or Acala SJ-2) [12], and for bacterial blight resistance in the BC_4_F_4_ generation within an intraspecific cross of cotton [13]. Transgressive segregation for resistance also has been reported in other plant-pathogen systems, such as soybean - soybean cyst nematodes [14], soybean - *Rhizoctonia* root and hypocotyl rot [15], Arabidopsis – *Leptosphaeria maculans*, blackleg disease [16], wheat – stripe rust [17]–[20], barley – *Pyrenophora teres* [21] and barley - powdery mildew [22].

Individual cotton plants highly resistant to *M. incognita* (transgressive segregants) have been reported within Auburn 623 RNR and some N-lines [23]–[25]. Turcotte et al. (1963) [26] reported that two recessive genes determined *M. incognita* resistance based on susceptible F_1_ and segregating F_2_ in a study of crosses of root-knot-resistant *G. darwinii* x susceptible *G. barbadense* cv. Pima S-1 and Pima S-2 breeding stocks. Genetic and quantitative trait loci (QTL) analyses revealed at least one major recessive gene with strong additive effect in intraspecific crosses Acala NemX x Acala SJ-2 and Acala SJ-2 x Clevewilt and one major QTL with strong dominant effect in the interspecific cross Pima S-7 x Acala NemX [8]–[9], [12], [26]. A major telomeric segment on chromosome 11 harbors RKN resistance genes from different backgrounds [5], [8], [27]–[29].

Transgressive segregation appears to be quite common in allotetraploid cotton, although the genetic basis of the generated novel phenotypes is generally not known. For traits that involve complex interactions of multiple genes, a QTL mapping approach can be informative for studying inheritance and gene action [27]. In particular, detection of minor “modifier” genes that may otherwise escape detection can be improved by QTL analysis [30]–[31]. Here we report on QTL mapping analysis to characterize the transgressive segregation for RKN resistance in a recombinant inbred line (RIL) population developed from an interspecific cross of susceptible Upland TM-1 (*G. hirsutum*) x susceptible Pima 3–79 (*G. barbadense*) with the goal to improve introgression of novel combinations of RKN resistance QTLs into elite cotton cultivars using susceptible genotypes. The specific approach was to phenotype the RIL population to identify QTLs associated with RKN resistance, and to study epistatic effects among QTL regions associated with RKN resistance.

## Materials and Methods

### Plant materials and Nematode resistance screening

Original seed source of a recombinant inbred line (RIL) population developed from an interspecific cross between *G. hirsutum* Upland TM-1 and *G. barbadense* Pima 3–79 [32] was provided by Drs. RJ Kohel and JZ Yu of USDA-ARS in College Station, TX. *G. hirsutum* Acala NemX and Acala N901 were included as resistant controls and Acala SJ-2 and *G. barbadense* Pima S7 as susceptible controls. The population was used previously for mapping microsatellite or simple sequence repeat (SSR) markers [33]–[34]. One hundred thirty-eight recombinant inbred lines (RILs) [6], parents, and controls were evaluated for nematode response in infection assays under controlled conditions in a greenhouse at University of California, Riverside, CA in two experiments using previously published protocols [9]. Five replicate plants of each RIL were screened in each test. Each of the parents and resistant and susceptible controls were also represented by five replicate plants in each test, which were randomly positioned between the randomized RILs to confirm uniformity of test conditions. The five sets of replicates were arranged on greenhouse benches in a randomized complete block design. Briefly, three-week-old seedlings were inoculated with approximately 50,000 eggs of *M. incognita* race 3 (isolate Project 77, from a cotton field in California). Air temperatures in the greenhouse were maintained between 28 and 35°C during the day and 24°C at night. Individual plants were scored for phenotype 60 days after inoculation. A 0–10 root-gall rating scale [35] ranging from no galling (0) to severe galling reaction (10) was used to evaluate resistance reaction to nematodes (root galling index - GI) [9]. For experiment 1, GI was recorded, and assays of egg production per root system and per gram fresh root were made by extracting eggs from the roots in NaOCl [36] (nematode egg production - EGR). In experiment 2, only GI was recorded.

### Marker and genetic linkage analysis

One thousand and fifty SSR markers with wide genome coverage [6], [8], [33]–[34], [37, CMD, www. cottonmarker.org] were tested on the RIL (Upland TM-1 x Pima 3–79) population for association with RKN resistance. PCR amplification of BNL, CIR, Gh, MUSB, MUCS, MUSS, and NAU cotton molecular markers was performed on a total volume of 15 µL containing 2 µL of DNA template (concentration 10 ng), following the protocol described by Ulloa et al. (2011) [6]. PCR products were separated for 4 to 5 hrs on a 3% super fine resolution (SFR™) agarose gel (Amresco, Solon, OH) containing 1X TBE at 90 volts, and were visualized by Alphaimager software (v. 5.5, Alpha Innotech Corporation, San Leandro, CA) after staining with ethidium bromide. Primer-pairs were scored if they resulted in discrete PCR banding patterns (amplicons) denoting a molecular marker. Informative bands were scored as present (+) or absent (−) for a dominant marker, and if alleles from both parents were identified, then the marker was scored as co-dominant. The genotypic ratio of 1∶1 was expected for both dominant and co-dominant markers.

The JoinMap^R^ version 4.0 [38] computer program was used to test for Chi-square goodness-of fit for expected versus observed genotypic ratios, and to develop the linkage groups for chromosomes. Logarithm of odds (LOD) scores of 3 to 14 were examined for each population using the Kosambi map function, and a maximum distance of 40 cM was used to determine linkage between any two markers. Linkage analyses on the RILs used DNA isolated from Fusarium wilt-phenotyped plants [6], together with a previously developed genetic linkage map [33], [37]. Twenty-three linkage groups from the 24 cotton chromosomes were developed. The linkage groups/chromosomes were developed with LOD>6 to obtain strong linkage between two anchored markers. We selected this LOD score to represent specific chromosome regions of the cotton genome.

### Data analysis

Phenotypic data were subjected to one-way analysis of variance (ANOVA). Fisher's Protected LSD test was used to compare the treatment means using SAS (SAS, ver. 9.1.3; SAS Institute, Cary, NC). Data for nematode egg production were transformed to Log_10_(x+1) for analysis (LogEGR). Root-galling index (GI), eggs per gram of root (nematode egg production - EGR), and LogEGR were used for quantitative trait loci (QTL) analyses.

### QTL analysis

QTL analyses were conducted on GI, EGR, and LogEGR using MapQTL 5.0 [39]. Single-marker analysis was conducted by using nonparametric mapping [Kruskal-Wallis analysis (K*)] test equivalent of the one-way analysis of variance [39]. We used the nonparametric analysis, because in this test, no assumptions are being made for the probability distribution(s) of the quantitative trait, and even if the data are distributed normally, the nonparametric test is often as powerful as parametric methods. In addition, the nonparametric test uses all markers genotyped on the population regardless of their linkages (tests each locus separately without the use of the linkage map). Threshold value for a marker-QTL was determined at *P*<0.1. Significant QTLs were determined with a more stringent *P*<0.005 for Kruskal-Wallis [39]. In order to verify the cumulative effects of QTL on the GI and EGR nematode response phenotypes, RILs were classified according to the number of alleles contributing to the resistance (favorable or positive alleles) present at each of the QTLs. Then QTLs were pyramided by combining data with favorable alleles (+, ++, +++, or ++++) and null RIL-genotypes (−,−−,−−− or −−−−). Class-specific means of GI and LogEGR and standard error were calculated for each genotypic class.

## Results

### Phenotype of parents

**Figure 1:**
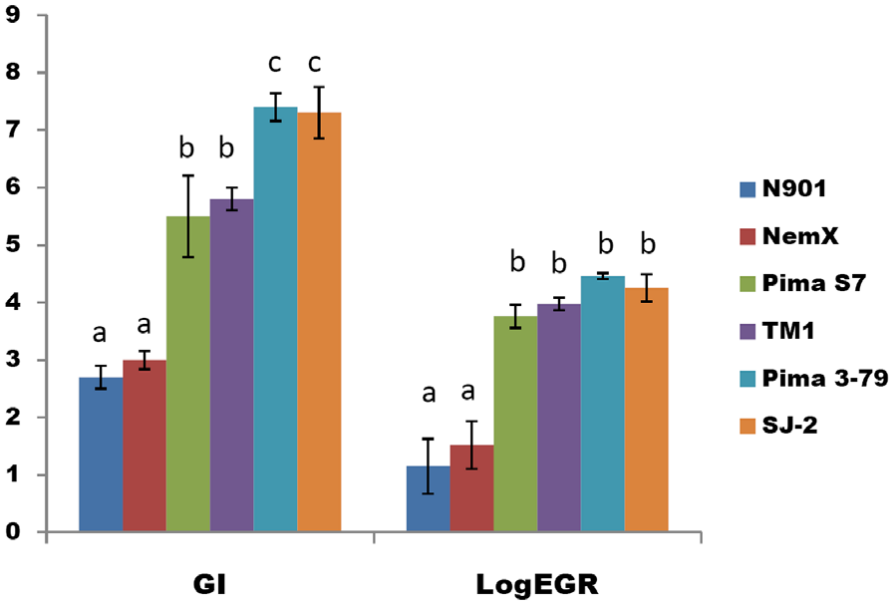
Root galling index (GI) and egg production (LogEGR) of *Meloidogyne incognita* on susceptible (TM-1, Pima 3–79, Pima S-7 and Acala SJ-2) and resistant (Acala N901 and Acala NemX) cotton cultivars. Data were collected 60 days after inoculation. The error bars show standard error.

Susceptible TM-1, Pima 3–79, Pima S7 and Acala SJ-2 had higher (*P*<0.05) galling indices (GI) and supported greater (*P*<0.05) numbers of eggs per gram root (EGR) than the two resistant genotypes Acala N901 (GI, 2.7, and LogEGR, 1.15) and Acala NemX (GI, 3, and LogEGR, 1.52) (Figure 1). Susceptible Pima S7 (GI, 5.4 and LogEGR, 3.76) and TM-1 (GI, 5.8 and LogEGR, 3.97) had lower (*P*<0.05) GI than the susceptible Pima 3–79 (GI 7.4, and LogEGR, 4.46) and SJ-2 (GI, 7.3 and LogEGR 4.25) (Figure 1).

### Phenotyping RILs

**Figure 2:**
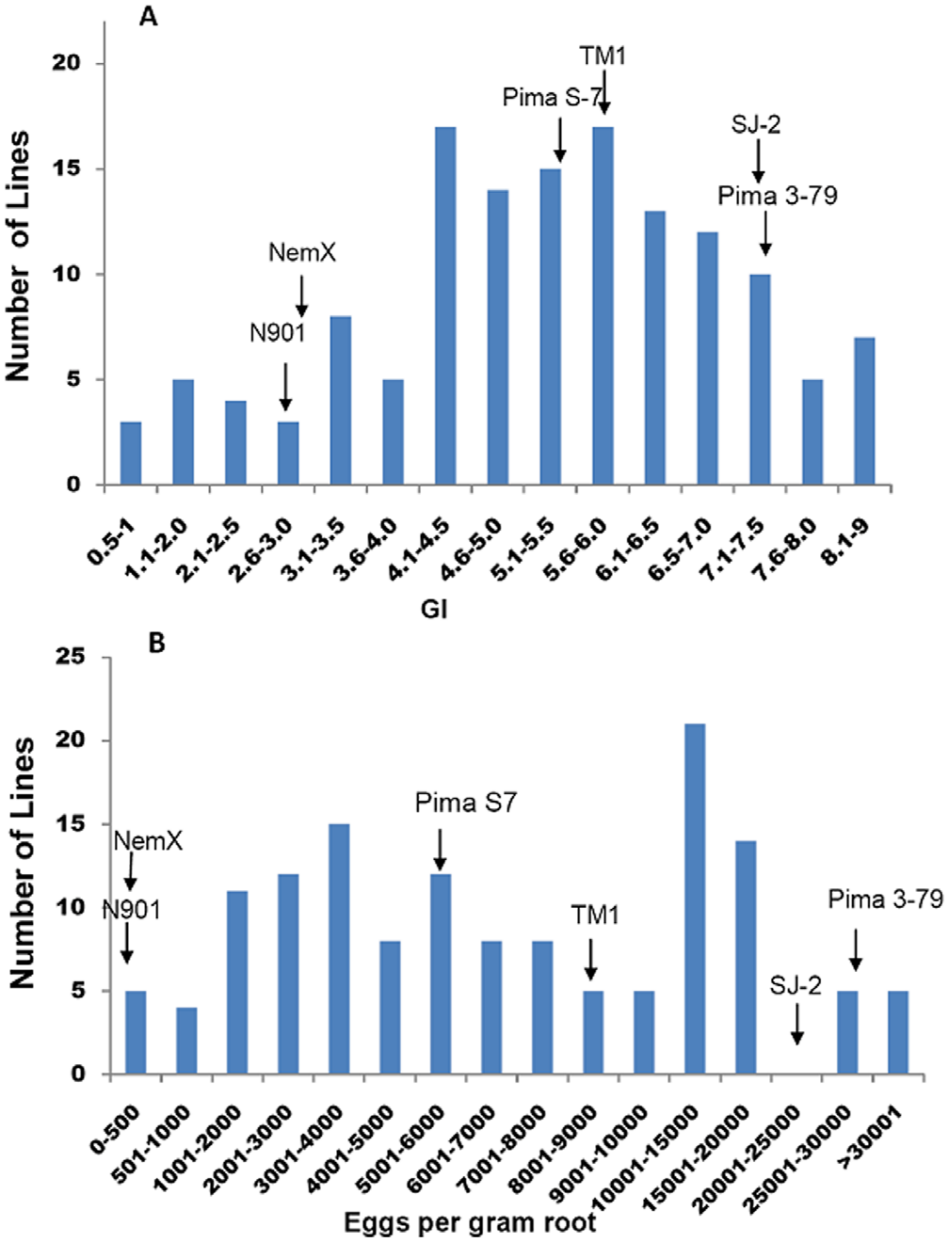
Distribution of galling index (a) and eggs per gram root (b) in the RIL (TM-1 x Pima 3–79) population. Data were collected 60 days after inoculation. Galling Index: 0–10 scale; 0 no galling, and 10 severe galling. The arrows point to the phenotypic reaction score in Acala N901, Acala NemX, Acala SJ-2, Pima S-7, TM-1 and Pima 3–79.

The RILs were phenotyped in the two greenhouse experiments (I and II). Different severity levels of infection were observed between the two experiments. Experiment II had less infection than Experiment I. This difference may have been caused by different environmental conditions in the greenhouse related to the season of the year during which each test was performed. Therefore, the phenotypic data are shown below for Experiment I. Seventy-four of 138 lines had lower (*P*<0.05) GI than the parent TM-1 (5.8±0.25 SE) indicating 53.6% of the lines exhibited transgressive resistance (Figure 2A), and 72 lines had fewer (*P*<0.05) EGR (8398±2025SE) indicating 52.1% of the lines showed transgressive resistance (Figure 2B). These observations indicated that multiple genes from both parents are involved in determining the resistance phenotype in these transgressive segregants because both parents are in the typical range of the susceptible phenotype for both GI and EGR. A low correlation (*R^2^* = 0.42) between GI and EGR in the RIL population indicated that different genes might control the GI and nematode reproduction responses.

### QTL mapping and identification

**Figure 3:**
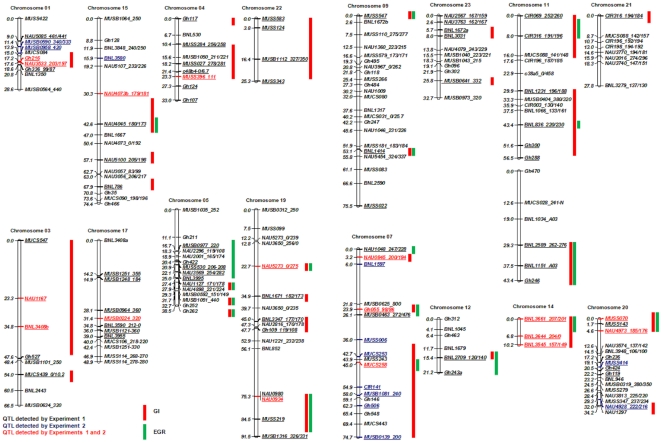
Map locations of QTLs associated with resistance to root-knot nematode. Single-marker analysis conducted using nonparametric mapping (Kruskal-Wallis analysis) test equivalent of the one-way analysis of variance (Van Ooijen 2004). Red bars - QTL influencing root-galling index phenotype; Green bars - QTL influencing egg production phenotype (For Experiment I).

Twenty-one linkage groups/chromosomes were identified and they were found to be involved in RKN transgressive resistance by QTL analyses in this study (Figure 3, Tables 1, S1, and S2). However, four highly significant (*P*<0.005) QTLs were revealed by the nonparametric mapping test analogous to one-way analysis of variance [39] on chromosomes 3 (*Mi-GI_h_ -C03_1_*, BNL3408), 4 (*Mi-GI_b_ -C04_1_*, MUSSS396_N-111), 11 (*Mi-GI_b_ -C11_1_*, BNL1231), and 17 (*Mi-GI_h_ -C17_1_*, MUSB0224_320) in Experiment I accounting for 12.3, 11.5, 10.1, and 7.95% of the phenotypic variance in GI, respectively (Table 1, Figure 3). Three of these QTLs were identified in both Experiment I and Experiment II even though weaker infection occurred in Experiment II (Figure 3), confirming that these QTLs contributed to transgressive segregation for resistance. Moreover, there were two significant (*P*<0.005) QTLs on chromosome 23 (*Mi-EGR_b_ -C23_1_*, BNL1672) and chromosome 14 (*Mi-EGR_h_ -C14_1_*, BNL3661) in Experiment I accounting for 10.6 and 9.7% of the phenotypic variance in nematode egg production, respectively (Table 1, Figure 3).

**Table 1:** QTLs associated by nonparametric mapping with root-galling index (GI) and nematode egg production (EGR) in TM-1 x Pima 3–79 RIL population.

QTL-GI^a^	Name	Chr^c^	Locus	K*^e^	Signif.^f^	TM-1 allele^g^	Pima 3–79 allele
1	*Mi-GI_h_-C03_1_* d	3	BNL3408b	12.32	******	4.88	5.93
2	*Mi-GI_h_-C17_1_*	17	MUSB0224/320	11.534	*****	4.83	5.83
3	*Mi-GI_b_-C04_1_*	4	MUSS396_111	10.071	****	5.73	4.84
4	*Mi-GI_b_-C11_1_*	11	BNL1231b_196/188	7.95	****	5.68	4.83
5	*Mi-GI_b_-C22_1_*	22	MUSB1112_327/350	7.744	***	5.66	4.88
6	*Mi-GI_b_-C05_1_*	5	Gh262	7.493	***	5.67	4.85
7	*Mi-GI_h_-C15_1_*	15	NAU4045_170/165	7.34	***	4.90	5.68
8	*Mi-GI_h_-C14_1_*	14	BNL3661_207/201^i^	7.103	***	4.94	5.71
9	*Mi-GI_b_-C20_1_*	20	Gh119	6.914	***	5.63	4.73
10	*Mi-GI_h_-C19_1_*	19	MUSS219	6.872	***	4.86	5.59
11	Mi-GI_h_-C07_1_	7	Gh055_98/86	6.315	**	5.09	5.96
12	*Mi-GI_b_-C12_1_*	12	MUSB0117_123/143	6.134	**	5.81	4.93
13	*Mi-GI_b_-C19_1_*	19	NAU5273_0/275	4.197	**	5.56	4.57
14	*Mi-GI_b_-C21_1_*	21	CIR316_194/184	4.041	**	5.65	4.95

QTL-EGR^b^							
1	*Mi-EGR_b_-C23_1_*	23	BNL1672a	10.644	****	3.93	3.63
2	*Mi-EGR_h_-C14_1_*	14	BNL3661_207/201	9.705	****	3.64	3.92
3	*Mi-EGR_h_-C24_1_*	24	NAU3605_222/227	7.279	***	3.63	3.86
4	*Mi-EGR_h_-C06_1_*	6	NAU4969_0/305	7.253	***	3.68	3.86
5	*Mi-EGR_h_-C15_1_*	15	NAU4045_180/173	6.244	**	3.68	3.86
6	*Mi-EGR_h_-C19_1_*	19	MUSS219	5.448	**	3.65	3.84
7	*Mi-EGR_b_-C11_1_*	11	CIR316_191/196	5.32	**	3.88	3.66
8	*Mi-EGR_h_-C12_1_*	12	Gh243a	4.862	**	3.66	3.86
9	*Mi-EGR_b_-C11_2_*	11	BNL2589_262/276	4.778	**	3.84	3.66

a QTL - Root-galling index (GI) phenotype;

b QTL- Data for nematode egg production were transformed to Log_10_(x+1) for analysis (Log EGR);

c Chr: Cotton chromosome designation;

d *Mi-GI_h_-C03_1_*: The name of first (1) identified QTL for GI on chr 3 from *G. hirsutum* (h) to root-knot nematode *Meloidogyne incognita* (*Mi*): *Mi-EGR_b_-C23*: The name of QTL for EGR on chr 23 from *G. barbadense (b)* to root-knot nematode *Meloidogyne incognita* (*Mi*);

e K*: Kruskal-Wallis analysis test regarded as the nonparametric equivalent of the one-way analysis of variance (Van Ooijen 2004);

f *P*-value: P-values are designated as P<0.05 (**), 0.01 (***), 0.005 (****), 0.001 (*****), 0.0005 (******);

g TM-1 **allele**: Mean value of phenotype associated with the TM-1 allele; Pima 3–79 allele, Mean value of phenotype associated with the Pima 3–79 allele.

Nineteen additional QTLs for GI and 15 for EGR were detected at *P*<0.05 (Table 1, Table S1). The map locations of some of the detected QTLs associated with resistance to RKN are shown in Figure 3. In total, eight pairs of homoeologous chromosomes (Chr 1–15, 3–17, 4–22, 5–19, 6–25, 8–24, 9–23 and 11–21) were involved in galling index and/or nematode production. Sixteen QTLs were involved in both GI and EGR (Figure 3, Table S2) in Experiment I. Two significant QTLs (*Mi-GI_h_ -C03_1_* and *Mi-GI_h_ -C17_1_*) were associated with GI but not with EGR. However, four significant QTLs (*Mi-GI_b_ -C11_1_, Mi-GI_b_ -C04_1_, Mi-EGR_h_ -C14_1_* and *Mi-EGR_b_ -C23_1_*) were involved in both GI and EGR (Figure 3, Table S2). Among 23 QTLs associated with GI, 10 had positive alleles from *G. hirsutum* TM-1 and 13 had positive alleles from *G. barbadense* Pima 3–79. Nine of 17 QTLs with positive alleles associated with EGR were from *G. hirsutum* TM-1 and 8 from *G. barbadense* Pima 3–79 (Table 1, Table S1).

**Figure 4:**
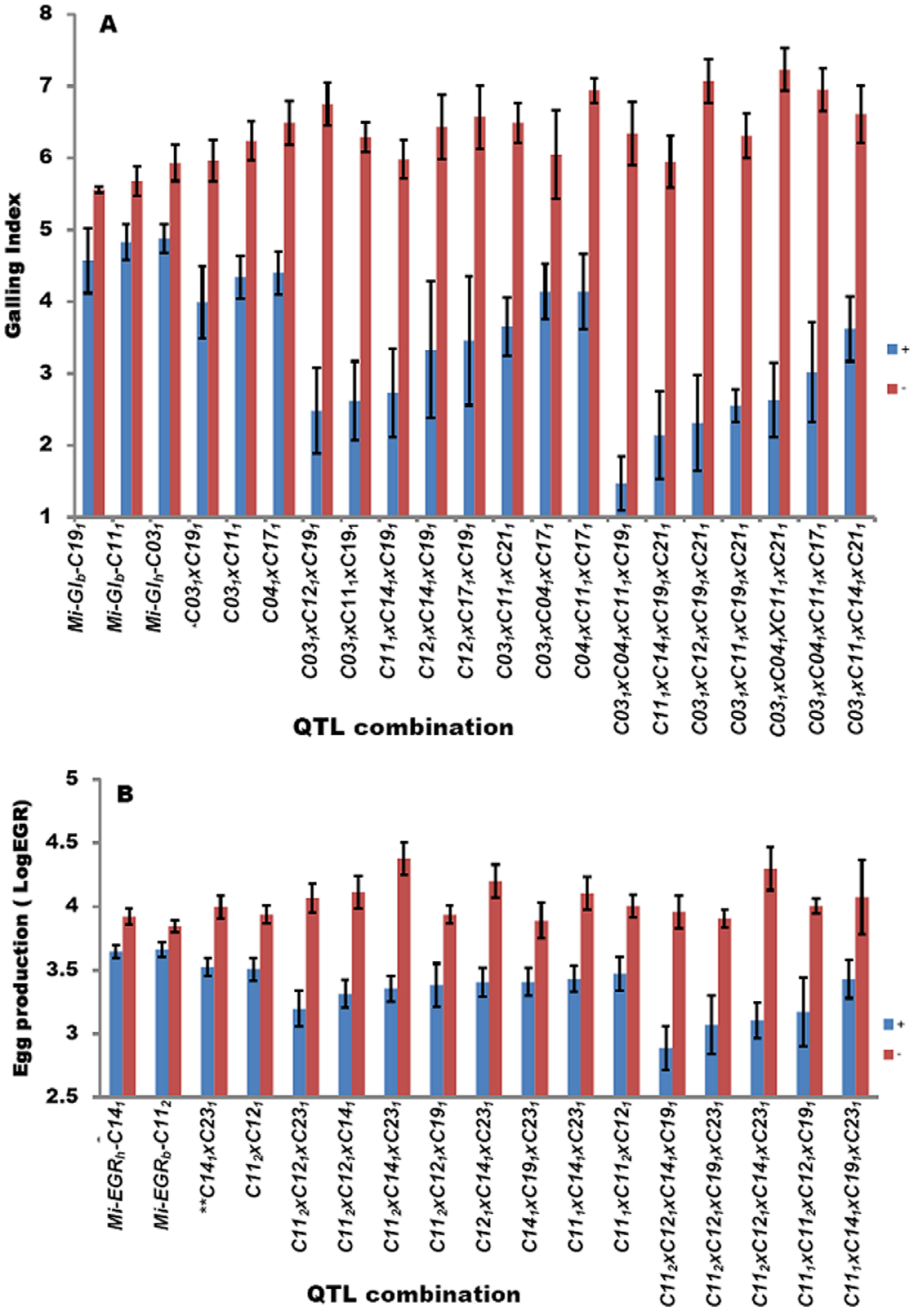
Effect of the combinations of QTLs for galling index and nematode egg production on mean phenotypic value (± standard error) based on genotypic classes carrying one to four favorable alleles (+, ++, +++, ++++ blue bars) and null genotypes (−,−−,−−−,−−−−, red bars) for Experiment I.

Analysis of QTL combinations with positive alleles contributed by both parents revealed that some putative detected QTLs (*P*<0.05) dramatically decreased GI when combined with major (*P*<0.005) QTLs or other putative QTLs. A few examples of these QTL combinations are represented in Figure 4. Combinations of the QTLs for nematode root-galling index and egg production were clearly associated with nematode infection response in the RIL population. The lines with a single positive QTL allele (+) had significantly lower GI and EGR (*P*<0.05; Figure 4). However, the combination of two or more QTLs with positive effect alleles resulted in much greater suppression of galling index and egg production (in some combinations more than 50% reduction) than those with only a single positive QTL allele (Figure 4, Table 1), suggesting additive or epistatic effects in those RIL-genotypes carrying two or more favorable alleles. Even though each single putative QTL could not contribute to high resistance to nematodes, highly resistant lines were formed when a few putative QTLs were combined together, such as the 3-QTL combinations *Mi-GI- C12_1_xC14_1_xC19_1_* (GI, 3.33) and *Mi-EGR-C11_2_xC12_1_xC19_1_* (LogEGR, 3.38) (Figure 4, Table 1) compared with parents TM-1 (GI, 5.8; LogEGR 3.97), Pima 3–79 (GI, 7.4; LogEGR 4.46) and resistant Acala NemX (GI, 3.0; LogEGR 1.52). We found that the response of root-galling suppression differed depending on selected QTLs. The 3-QTL combination of *Mi-GI-03_1_xC12_1_xC19_1_* (GI, 2.48) conferred a lower (*P*<0.05) galling index than the combination of three significant (*P*<0.05) QTLs *Mi-GI- C03_1_xC04_1_xC17 _1_* (GI, 4.14) or *Mi-GI-C04_1_xC11_1_xC17_1_* (GI, 4.14). The 4-QTL combination of *Mi-GI-C03_1_xC04_1_xC11_1_xC19_1_* (GI, 1.47) conferred a lower (*P*<0.05) galling index than the combination of four significant (*P*<0.05) QTLs *Mi-GI-C03_1_xC04_1_xC11_1_xC17_1_* (3.02) (Figure 4). Some combinations were observed to be better than others. For example, the combination of *Mi-EGR-C11_2_xC12_1_xC14_1_xC19_1_* resulted in less (*P*<0.05) egg production (LogEGR, 2.89) than that conferred by the combination *Mi-EGR-C11_1_xC14_1_xC19_1_xC23_1_* (LogEGR, 3.43). However, both combinations conferred lower eggs per root than the parents TM-1 (LogEGR, 3.97) and Pima 3–79 (LogEGR, 4.46). These epistatic effects in progeny derived from susceptible parents indicate that pyramiding of these QTLs presents a new level of nematode resistance in cotton.

## Discussion

Our QTL analyses of root-knot nematode resistance in a cotton RIL population developed from an interspecific cross demonstrates the extent to which transgressive segregation for nematode resistance can occur in interspecific progeny derived from plant types with standard susceptible phenotypes. Previously we documented transgressive segregation for resistance in progenies derived from interspecific parents in which one of the parents carried a known major gene for resistance, *rkn1* on chromosome 11 [8]. In that case, the susceptible parent was found to contribute a gene, *RKN2*, that had an undetectable effect on nematode response phenotype in the susceptible parent Pima S-7. However, presence of both genes resulted in extremely high resistance beyond that determined by the major gene alone and indicated an epistatic interaction that strengthened the resistance phenotype. We had also discovered in an intraspecific *G. hirsutum* cross between an *rkn1* parent (Acala NemX) and a susceptible genotype (Acala SJ-2) in which some progenies exceeded the resistance level of the resistant parent [9]. That work built on an earlier report by Shepherd (1974) [23] of transgressive segregants for nematode resistance in progenies developed from a cross of two partially resistant intraspecific *G. hirsutum* cotton lines (Clevewilt and Wild Mexico Jack Jones). In the latter case, recent analysis of derived lines indicated that a combination of two genes, one from each parent, residing on chromosomes 11 and 14, were responsible for the combined resistance phenotypes [40].

In the current study, even considering this range of documented transgressive segregation events for resistance in cotton, the extent of resistance expression in the progenies generated from susceptible parents was remarkable. We found that more than 50% of recombinant inbred lines were more resistant than either susceptible parent. Importantly, in some lines the levels of resistance measured by suppression of root-galling and (or) nematode egg production were higher than standard resistant control lines such as Acala NemX included in the phenotyping screens. The QTL analysis revealed 23 major and putative QTLs involved in suppression of root-galling and 17 for suppressing egg production, indicating high transgressive segregation ratios. Obviously, the combinations of positive allele QTLs existing in each parent are not effective in conferring nematode resistance. Rather, only combinations of certain positive alleles contributed from both parents provided the unique genotypes required for nematode resistance. Thus, a single QTL had low effect on galling index and egg production, but when combined in positive allele forms, higher resistance levels were conferred (Figure 4 and Table 1), including combinations involving some of the putative small-effect QTLs. The analysis of effect on phenotype of various combinations of up to several QTLs revealed that some combinations of four QTL could produce high levels of nematode resistance for both root-galling and egg production phenotypes. This is apparent from the comparisons of the phenotypic averages of lines with or without the positive alleles for the specific QTL combination (Figure 4). These small-set QTL combinations could provide the basis for effective plant selection targets for genetic improvement in cotton breeding programs.

QTL analysis indicated that there were eight homoeologous chromosomes involved in the root-galling index (GI) and/or nematode egg production (EGR) phenotypes. Chromosome 11 of cotton is rich in disease resistance genes, including resistance to root-knot nematode [8], [29], [40]–[43], reniform nematode [44]–[45], Fusarium wilt [6], Verticillium wilt [10] and black root rot [46]. The marker CIR316-191/196 on chromosome 11 was involved in both GI and EGR, supporting our previous finding with gene *rkn1* that resistance genes could suppress both root-galling and egg production [8]–[9]. The marker CIR316-194/184 on chromosome 21 also contributed to decreasing root-galling indicating some homological gene region on the paired homoeologous chromosome 11. These two chromosomes (11 and 21) might share gene expression functions in cotton. Ulloa et al. (2010) [27] reported that minor genes on chromosome 21 associated with markers CIR316 and MUCS088 contributed to both egg production and root-galling phenotypes in the F_2_ population of Pima S-7 x Acala NemX. A minor gene on chromosome 21 was also identified to contribute to suppressing root-galling in the F_2_ and F_7_ populations of Acala NemX and Acala SJ-2 (NxS) and BC_1_F_1_ (Acala SJ-2 x F_1_ (NxS) (Wang and Roberts, unpublished data). The homoeologous chromosome pair 11/21 also may be involved in reniform nematode resistance based on genetic mapping studies [44]–[45], [47]–[48]. Depending on populations, the regions involved in both root-knot nematode and reniform nematode resistance are around 30 cM apart, based on linked markers [48]. The sequencing of markers in these regions associated with both chromosomes 11 and 21 is underway and should help in resolving the complexity of genome recombination between the pair of homoeologous chromosomes. In the current study marker BNL3661 on chromosome 14 contributed more to inhibiting egg production than to suppressing root-galling, with the positive allele derived from *G. hirsutum* TM-1. This result is consistent with the report that a gene in this region from *G. hirsutum* M240 contributed to reduced egg production [40]. The marker BNL3545 close to BNL3661 on chromosome 14 involved in reducing egg production was also identified with the F_2_ population of Acala NemX x Acala SJ-2 (Wang and Roberts, unpublished data). Our finding that 14 QTLs were involved in both GI and EGR suggested that the relative contributions of these genes to both root-galling and egg production phenotypes are complex. Sequencing these QTL regions and comparing those sequences with other plants would expedite molecular breeding in cotton and enhance understanding of cotton evolution. Cotton whole genome sequencing currently in progress (http://www.monsanto.com/newsviews/Pages/Monsanto-Illumina-Key-Milestone-Cotton-Genome-Sequencing.aspx) will provide critical information about the cotton genome, plant evolution and adaption as it relates to valuable processes including transgressive segregation.

Our finding that susceptible parents can produce highly resistant progenies has important potential for exploitation in plant breeding. While transgressive segregation for biotic and abiotic stress tolerance and resistance is not uncommon, we have found very few reports where such highly resistant novel genotypes have been identified. Traditionally, crosses are made between a known resistance donor parent and susceptible parent with favorable traits, or between two moderately resistant parents, and the most resistant progeny are selected for advancement. Wallwork and Johnson (1984) [49] reported in wheat that 10% (24/240) of lines in a F_2_ population and 21.2% of lines (50/235) in a F_3_ population showed more resistance to yellow rust than either susceptible parent. Welch and Rieseberg (2002) [50] and Lexer et al. [51] reported that progenies showed 5–14 times more salt tolerance than the parental species in wild sunflower. Both sensitivity to salt and corresponding QTLs involved in salt tolerance were mapped to the chromosome level and used to select salt tolerant genotypes [52]. Based on the current findings, we suggest that efforts to identify novel and valuable phenotypes for biotic and abiotic stress resistance traits among progenies developed from stress susceptible or sensitive parent combinations are worthy of increased attention in plant breeding programs. This work also adds further support to the importance of outcrossing events in natural populations which drive selective advantage, adaptation and speciation processes.

## Supporting Information

Table S1 **QTLs associated by nonparametric mapping with root-galling and nematode egg production in TM-1 x Pima 3-79 RIL population.**
(DOC)

Table S2 **QTLs associated by nonparametric mapping with both root-galling and nematode egg production with same maker locus in TM-1 x Pima 3-79 RIL population.**
(DOC)
