# Supplementary material for: QTL Analysis for Transgressive Resistance to Root-Knot Nematode in Interspecific Cotton (Gossypium spp.) Progeny Derived from Susceptible Parents
Source: PLoS One. 2012 Apr 13;7(4):e34874. doi: 10.1371/journal.pone.0034874 (PMC3325951; doi:10.1371/journal.pone.0034874)
Supplement: Table S1 — QTLs associated by nonparametric mapping with root-galling and nematode egg production in TM-1 x Pima 3-79 RIL population. (DOC) [file pone.0034874.s001.doc]

Supplemental Data

**Table S1** QTLs associated by nonparametric mapping with root-galling and nematode egg production in TM-1 x Pima 3-79 RIL population

| QTL-GI^a^ | Name | Chr ^c^ | Locus | K*^e^ | Signif.^f^ | TM1  allele^g^ | Pima 3-79 allele |
| --- | --- | --- | --- | --- | --- | --- | --- |
| 15 | *Mi-GI_h_-C25_1_^d^* | 25 | MUSB1035-290 | 6.522 | ** | 5.44 | 4.06 |
| 16 | *Mi-GI_h_-C24_1_* | 24 | NAU3605_222/227 | 6.447 | ** | 4.77 | 5.73 |
| 17 | *Mi-GI_h_-C12_1_* | 12 | MUSS101_279-277 | 5.928 | ** | 4.82 | 5.74 |
| 18 | *Mi-GI_b_-C13_1_* | 13 | NAU1201 | 5.588 | ** | 5.60 | 4.81 |
| 19 | *Mi-GI_b_-C09_1_* | 9 | NAU3967_251/0 | 5.087 | ** | 5.95 | 5.08 |
| 21 | *Mi-GI_b_-C23_1_* | 23 | BNL1672a | 4.648 | ** | 5.73 | 4.94 |
| 20 | *Mi-GI_b_-C24_1_* | 24 | NAU1037_213/221 | 4.475 | ** | 5.52 | 4.78 |
| 22 | *Mi-GI_b_-C08_1_* | 8 | BNL3257_217/196 | 4.459 | ** | 5.58 | 4.92 |
| 23 | *Mi-GI_h_-C01_1_* | 1 | Gh216 | 4.447 | ** | 4.89 | 5.63 |
| QTL-EGR^b^ |  |  |  |  |  |  |  |
| 10 | *Mi-EGR_b_-C05_1_* | 5 | MUSB0977_220 | 6.4 | ** | 3.87 | 3.69 |
| 11 | *Mi-EGR_b_-C09_1_* | 9 | NAU3967_251/0 | 6.141 | ** | 3.97 | 3.70 |
| 12 | *Mi-EGR_b_-C20_1_* | 20 | Gh424 | 5.755 | ** | 3.83 | 3.65 |
| 13 | *Mi-EGR_h_-C05_1_* | 5 | MUCS045_351/357 | 5.444 | ** | 3.66 | 3.85 |
| 14 | *Mi-EGR_h_-C13_1_* | 13 | BNL0409_90 | 5.131 | ** | 3.67 | 3.85 |
| 15 | *Mi-EGR_b_-C04_1_* | 4 | MUSS396_111 | 4.726 | ** | 3.83 | 3.68 |
| 16 | *Mi-EGR_h_-C19_2_* | 19 | Gh209 | 4.296 | ** | 3.69 | 3.83 |
| 17 | *Mi-EGR_b_-C08_1_* | 8 | MUSB1188_1.25kb | 4.223 | ** | 3.83 | 3.70 |

**a** QTL - Root-galling index (GI) phenotype;

**b** QTL- Data for nematode egg production were transformed to Log_10_(x+1) for analysis (Log EGR);

**c** Chr: Cotton chromosome designation;

**d** *Mi-GI_h_-C25_1_*: The name of first (1) identified QTL for GI on chr 25 from *G. hirsutum* (h) to root-knot nematode *Meloidogyne incognita* (*Mi*): *Mi-EGR_b_-C05_1_:* The name of QTL for EGR on chr 23 from *G. barbadense (b)* to root-knot nematode *Meloidogyne incognita* (*Mi*);

**e** K*: Kruskal-Wallis analysis test regarded as the nonparametric equivalent of the one-way analysis of variance (Van Ooijen 2004);

**f** *P* value: P values are designated as *P* <0.05 (**), 0.01 (***), 0.005 (****), 0.001 (*****), 0.0005 (******);

**g** TM-1 **allele**: Mean value of phenotype associated with the TM-1 allele; Pima 3-79 allele, Mean value of phenotype associated with the Pima 3-79 allele.
