# Supplementary material for: QTL Analysis for Transgressive Resistance to Root-Knot Nematode in Interspecific Cotton (Gossypium spp.) Progeny Derived from Susceptible Parents
Source: PLoS One. 2012 Apr 13;7(4):e34874. doi: 10.1371/journal.pone.0034874 (PMC3325951; doi:10.1371/journal.pone.0034874)
Supplement: Table S2 — QTLs associated by nonparametric mapping with both root-galling and nematode egg production with same maker locus in TM-1 x Pima 3-79 RIL population. (DOC) [file pone.0034874.s002.doc]

Supplemental Data

**Table S2** QTLs associated by nonparametric mapping with both root-galling and nematode egg production with same maker locus in TM-1 x Pima 3-79 RIL population

| # | Trait^a^ | Name of QTL ^b^ | Chr ^c^ | Locus | K*^d^ | Signif.^e^ | TM1  allele^f^ | Pima 3-79 allele |
| --- | --- | --- | --- | --- | --- | --- | --- | --- |
| 1 | GI | Mi_1b_-GI-C04 | 4 | MUSS396_ 111 | 10.071 | **** | 5.72918 | 4.83591 |
|  | EGR |  | 4 | MUSS396_ 111 | 4.715 | ** | 10593.8 | 7346.64 |
|  | LogEGR | *Mi-EGR_b_-C04_1_* | 4 | MUSS396_ 111 | 4.726 | ** | 3.82884 | 3.67932 |
|  | GI |  | 4 | Gh117 | 7.848 | *** | 5.67684 | 4.77878 |
|  | EGR |  | 4 | Gh117 | 3.026 | * | 10791.4 | 7797.63 |
|  | LogEGR |  | 4 | Gh117 | 3.026 | * | 3.82949 | 3.70871 |
|  | GI |  | 4 | Gh124 | 7.823 | *** | 5.71852 | 4.90569 |
|  | EGR |  | 4 | Gh124 | 2.931 | * | 10569.5 | 8251.5 |
|  | LogEGR |  | 4 | Gh124 | 2.931 | * | 3.83708 | 3.7034 |
|  |  |  |  |  |  |  |  |  |
| 2 | GI | *Mi-GI_b_-C05_1_* | 5 | NAU1172 | 5.767 | ** | 5.74761 | 5.02829 |
|  | EGR |  | 5 | NAU1172 | 3.617 | * | 10420.8 | 8388.66 |
|  | LogEGR | *Mi-EGR_b_-C05_1_* | 5 | NAU1172 | 3.647 | * | 3.83178 | 3.70882 |
|  |  |  |  |  |  |  |  |  |
| 3 | GI | *Mi-GI_b_-C08_1_* | 8 | MUSB1188_1.25kb | 3.2 | * | 5.54981 | 5.04974 |
|  | EGR |  | 8 | MUSB1188_1.25kb | 4.223 | ** | 10987.9 | 7771.43 |
|  | LogEGR | *Mi-EGR_b_-C08_1_* | 8 | MUSB1188_1.25kb | 4.223 | ** | 3.83485 | 3.70027 |
|  | GI |  | 8 | MUSB0662_190/178 | 2.716 | * | 5.6268 | 5.15315 |
|  | EGR |  | 8 | MUSB0662_190/178 | 3.91 | ** | 11293.7 | 7924.03 |
|  | LogEGR |  | 8 | MUSB0662_190/178 | 3.899 | ** | 3.84624 | 3.71007 |
|  | GI |  | 8 | MUCS148 | 2.98 | * | 5.61936 | 5.13377 |
|  | EGR |  | 8 | MUCS148 | 3.631 | * | 11580.9 | 7626.46 |
|  | LogEGR |  | 8 | MUCS148 | 3.619 | * | 3.8454 | 3.72059 |
|  | GI |  | 8 | MUCS248 | 2.923 | * | 5.59152 | 5.06627 |
|  | EGR |  | 8 | MUCS248 | 3.303 | * | 11350.5 | 7402.4 |
|  | LogEGR |  | 8 | MUCS248 | 3.293 | * | 3.8305 | 3.70663 |
|  | GI |  | 8 | MUSB0073_700/620 | 2.864 | * | 5.65595 | 5.014 |
|  | EGR |  | 8 | MUSB0073_700/620 | 2.756 | * | 11148.9 | 7948.93 |
|  | LogEGR |  | 8 | MUSB0073_700/620 | 2.743 | * | 3.86241 | 3.73524 |
|  | GI |  | 8 | NAU1046_689/0 | 2.776 | * | 5.55938 | 5.0403 |
|  | EGR |  | 8 | NAU1046_689/0 | 2.813 | * | 10841.6 | 7482.93 |
|  | LogEGR |  | 8 | NAU1046_689/0 | 2.806 | * | 3.82983 | 3.69401 |
|  | GI |  | 8 | NAU3769_0/289 | 2.938 | * | 5.51197 | 5.06672 |
|  | EGR |  | 8 | NAU3769_0/289 | 2.795 | * | 10765.9 | 7616.61 |
|  | LogEGR |  | 8 | NAU3769_0/289 | 2.787 | * | 3.82411 | 3.70122 |
|  |  |  |  |  |  |  |  |  |
| 4 | GI | *Mi-GI_b_-C09_1_* | 9 | NAU3967_251/0 | 5.087 | ** | 5.94567 | 5.08475 |
|  | EGR |  | 9 | NAU3967_251/0 | 6.155 | ** | 13559.8 | 7932.1 |
|  | LogEGR | *Mi-EGR_b_-C09_1_* | 9 | NAU3967_251/0 | 6.141 | ** | 3.96797 | 3.70161 |
|  |  |  |  |  |  |  |  |  |
| 5 | GI | *Mi-GI_b_-C11_1_* | 11 | CIR069_252/260 | 4.341 | ** | 5.74239 | 5.00579 |
|  | EGR |  | 11 | CIR069_252/260 | 6.155 | ** | 11604.5 | 7058.75 |
|  | LogEGR | *Mi-EGR_b_-C11_1_* | 11 | CIR069_252/260 | 6.172 | ** | 3.90026 | 3.6674 |
|  | GI |  | 11 | BNL836_220/230 | 6.33 | ** | 5.68476 | 4.85949 |
|  | EGR |  | 11 | BNL836_220/230 | 3.901 | ** | 9432.25 | 9200.2 |
|  | LogEGR |  | 11 | BNL836_220/230 | 3.891 | ** | 3.84846 | 3.68081 |
|  | GI |  | 11 | CIR316_191/196 | 3.799 | * | 5.62576 | 4.99415 |
|  | EGR |  | 11 | CIR316_191/196 | 5.308 | ** | 11048.8 | 7556.6 |
|  | LogEGR |  | 11 | CIR316_191/196 | 5.32 | ** | 3.87598 | 3.66254 |
|  |  |  |  |  |  |  |  |  |
| 6 | GI | *Mi-GI_b_-C11_2_* | 11 | BNL2589_262_276 | 3.576 | * | 5.51111 | 4.96984 |
|  | EGR |  | 11 | BNL2589_262_276 | 4.746 | ** | 10401.2 | 7431.37 |
|  | LogEGR | *Mi-EGR_b_-C11_2_* | 11 | BNL2589_262_276 | 4.778 | ** | 3.84437 | 3.66171 |
|  |  |  |  |  |  |  |  |  |
| 7 | GI | *Mi-GI_h_-C12_1_* | 12 | BNL2709_120/140c12 | 4.413 | ** | 4.90477 | 5.68185 |
|  | EGR |  | 12 | BNL2709_120/140c12 | 2.72 | * | 8244.09 | 9876.81 |
|  | LogEGR | *Mi-EGR_h_-C12_1_* | 12 | BNL2709_120/140c12 | 2.72 | * | 3.67411 | 3.8365 |
|  |  |  |  |  |  |  |  |  |
| 8 | GI | *Mi-GI_h_-C14_1_* | 14 | BNL3661_207/201 | 7.103 | *** | 4.94301 | 5.7104 |
|  | EGR |  | 14 | BNL3661_207/201 | 9.705 | **** | 7106.95 | 11776.4 |
|  | LogEGR | *Mi-EGR_h_-C14_1_* | 14 | BNL3661_207/201 | 9.705 | **** | 3.6447 | 3.9207 |
|  |  |  |  |  |  |  |  |  |
| 9 | GI | *Mi-GI_h_-C15_1_* | 15 | NAU4045_170/165 | 7.34 | *** | 4.89867 | 5.68033 |
|  | EGR |  | 15 | NAU4045_170/165 | 5.298 | ** | 8075.72 | 10331.7 |
|  | LogEGR | *Mi-EGR_h_-C15_1_* | 15 | NAU4045_170/165 | 5.286 | ** | 3.67447 | 3.85164 |
|  | GI |  | 15 | NAU5100_205/198 | 2.942 | * | 5.09534 | 5.53684 |
|  | EGR |  | 15 | NAU5100_205/198 | 2.909 | * | 8454.34 | 9973.96 |
|  | LogEGR |  | 15 | NAU5100_205/198 | 2.909 | * | 3.70275 | 3.83184 |
|  |  |  |  |  |  |  |  |  |
| 10 | GI | *Mi-GI_b_-C19_1_* | 19 | NAU5273_0/275 | 4.197 | ** | 5.55557 | 4.56522 |
|  | EGR |  | 19 | NAU5273_0/275 | 4.483 | ** | 9507.86 | 7043.74 |
|  | LogEGR | *Mi-EGR_b_-C19_1_* | 19 | NAU5273_0/275 | 4.467 | ** | 3.82759 | 3.60457 |
|  |  |  |  |  |  |  |  |  |
| 11 | GI | *Mi-GI_h_-C19_1_* | 19 | MUSS219 | 6.872 | *** | 4.86055 | 5.58855 |
|  | EGR |  | 19 | MUSS219 | 5.459 | ** | 8481.71 | 9565.26 |
|  | LogEGR | *Mi-EGR_h_-C19_1_* | 19 | MUSS219 | 5.448 | ** | 3.64967 | 3.83692 |
|  | GI |  | 19 | MUSB1316_326/331 | 5.714 | ** | 4.94985 | 5.61387 |
|  | EGR |  | 19 | MUSB1316_326/331 | 5.816 | ** | 8180.57 | 9892.84 |
|  | LogEGR |  | 19 | MUSB1316_326/331 | 5.839 | ** | 3.66731 | 3.84224 |
|  | GI |  | 19 | NAU0934b | 3.821 | * | 5.02426 | 5.53545 |
|  | EGR |  | 19 | NAU0934b | 3.506 | * | 8573.25 | 9491.18 |
|  | LogEGR |  | 19 | NAU0934b | 3.524 | * | 3.68367 | 3.82792 |
|  | GI |  | 19 | NAU0980 | 3.821 | * | 5.02426 | 5.53545 |
|  | EGR |  | 19 | NAU0980 | 3.506 | * | 8573.25 | 9491.18 |
|  | LogEGR |  | 19 | NAU0980 | 3.524 | * | 3.68367 | 3.82792 |
|  |  |  |  |  |  |  |  |  |
| 12 | GI |  | 20 | BNL946 | 2.871 | * | 5.5015 | 4.97944 |
|  | EGR |  | 20 | BNL946 | 2.996 | * | 10756.6 | 6793.7 |
|  | LogEGR |  | 20 | BNL946 | 2.996 | * | 3.81522 | 3.67793 |
|  | GI | *Mi-GI_b_-C20_1_* | 20 | Gh119 | 6.914 | *** | 5.62524 | 4.72553 |
|  | EGR |  | 20 | Gh119 | 4.048 | ** | 10922.3 | 6703.5 |
|  | LogEGR | *Mi-EGR_b_-C20_1_* | 20 | Gh119 | 4.062 | ** | 3.85127 | 3.66279 |
|  | GI |  | 20 | Gh424 | 6.382 | ** | 5.63333 | 4.89246 |
|  | EGR |  | 20 | Gh424 | 5.731 | ** | 11039 | 6347.23 |
|  | LogEGR |  | 20 | Gh424 | 5.755 | ** | 3.83295 | 3.64796 |
|  | GI |  | 20 | MUSB0319_380/350c20S | 4.228 | ** | 5.58836 | 4.98094 |
|  | EGR |  | 20 | MUSB0319_380/350c20S | 3.53 | * | 10841.5 | 7269.34 |
|  | LogEGR |  | 20 | MUSB0319_380/350c20S | 3.54 | * | 3.82822 | 3.68398 |
|  | GI |  | 20 | MUSS070 | 4.803 | ** | 5.69456 | 5.05095 |
|  | EGR |  | 20 | MUSS070 | 2.903 | * | 10862.2 | 7925.09 |
|  | LogEGR |  | 20 | MUSS070 | 2.895 | * | 3.85235 | 3.70538 |
|  | GI |  | 20 | NAU4973_185/176 | 3.128 | * | 5.59867 | 5.04349 |
|  | EGR |  | 20 | NAU4973_185/176 | 3.19 | * | 10739.2 | 7751.95 |
|  | LogEGR |  | 20 | NAU4973_185/176 | 3.181 | * | 3.85398 | 3.68511 |
|  |  |  |  |  |  |  |  |  |
| 13 | GI | *Mi-GI_b_-C22_1_* | 22 | MUSB1112_327/350 | 7.744 | *** | 5.66217 | 4.87559 |
|  | EGR |  | 22 | MUSB1112_327/350 | 4.725 | ** | 11316.7 | 7166.39 |
|  | LogEGR | *Mi-EGR_b_-C22_1_* | 22 | MUSB1112_327/350 | 4.702 | ** | 3.84282 | 3.69275 |
|  |  |  |  |  |  |  |  |  |
| 14 | GI | *Mi-GI_b_-C23_1_* | 23 | BNL1672a | 4.648 | ** | 5.73062 | 4.93706 |
|  | EGR |  | 23 | BNL1672a | 10.607 | **** | 12780.7 | 6908.24 |
|  | LogEGR | *Mi-EGR_b_-C23_1_* | 23 | BNL1672a | 10.644 | **** | 3.92879 | 3.63265 |
|  | GI |  | 23 | NAU3763_162/167 | 4.019 | ** | 5.72462 | 4.99324 |
|  | EGR |  | 23 | NAU3763_162/167 | 7.033 | *** | 12275.4 | 7308.39 |
|  | LogEGR |  | 23 | NAU3763_162/167 | 7.061 | *** | 3.90558 | 3.67013 |
|  |  |  |  |  |  |  |  |  |
| 15 | GI | *Mi-GI_h_-C24_1_* | 24 | NAU3605_222/227 | 6.447 | ** | 4.77318 | 5.73328 |
|  | EGR |  | 24 | NAU3605_222/227 | 7.296 | *** | 7318.59 | 10618.5 |
|  | LogEGR | *Mi-EGR_h_-C24_1_* | 24 | NAU3605_222/227 | 7.279 | *** | 3.63489 | 3.86047 |
|  |  |  |  |  |  |  |  |  |
| 16 | GI | *Mi-GI_h_-C25_1_* | 25 | MUSB1035_290 | 6.522 | ** | 5.44237 | 4.06143 |
|  | EGR |  | 25 | MUSB1035_290 | 3.225 | * | 9573.53 | 5455.71 |
|  | LogEGR | *Mi-EGR-C25_1_* | 25 | MUSB1035_290 | 3.225 | * | 3.79029 | 3.51314 |

**a** QTL - Root-galling index (GI) phenotype; QTL- Data for nematode egg production (EGR) were transformed to Log_10_(x+1) for analysis (Log EGR);

**b** *Mi-GI_h_-C25_1_*: The name of first (1) identified QTL for GI on chr 25 from *G. hirsutum* (h) to root-knot nematode *Meloidogyne incognita* (*Mi*): *Mi-EGR_b_-C05_1_:* The name of QTL for EGR on chr 23 from *G. barbadense (b)* to root-knot nematode *Meloidogyne incognita* (*Mi*);

**c** Chr: Cotton chromosome designation;

**d** K*: Kruskal-Wallis analysis test regarded as the nonparametric equivalent of the one-way analysis of variance (Van Ooijen 2004);

**e***P* value: *P* values are designated as *P* <0.1 (*), 0.05 (**), 0.01 (***), 0.005 (****), 0.001 (*****), 0.0005 (******);

**f** TM-1 allele: Mean value of phenotype associated with the TM-1 allele; Pima 3-79 allele, Mean value of phenotype associated with the Pima 3-79 allele.
